# S100A8/S100A9 cytokine acts as a transcriptional coactivator during breast cellular transformation

**DOI:** 10.1126/sciadv.abe5357

**Published:** 2021-01-01

**Authors:** Ruisheng Song, Kevin Struhl

**Affiliations:** Department of Biological Chemistry and Molecular Pharmacology, Harvard Medical School Boston, MA 02115, USA.

## Abstract

Cytokines are extracellular proteins that convey messages between cells by interacting with cognate receptors at the cell surface and triggering signaling pathways that alter gene expression and other phenotypes in an autocrine or paracrine manner. Here, we show that the calcium-dependent cytokines S100A8 and S100A9 are recruited to numerous promoters and enhancers in a model of breast cellular transformation. This recruitment is associated with multiple DNA sequence motifs recognized by DNA binding transcription factors that are linked to transcriptional activation and are important for transformation. The cytokines interact with these transcription factors in nuclear extracts, and they activate transcription when artificially recruited to a target promoter. Nuclear-specific expression of S100A8/A9 promotes oncogenic transcription and leads to enhanced breast transformation phenotype. These results suggest that, in addition to its classical cytokine function, S100A8/A9 can act as a transcriptional coactivator.

## Teaser

S100A8/S100A9, a secreted cytokine, also acts in the nucleus as a transcriptional coactivator to promote cellular transformation.

## INTRODUCTION

Cytokines are a large and diverse group of small proteins that are important in many biological processes, such as immune responses, inflammation, and cancer. By conveying messages between cells by interacting with their cognate receptors at the cell surface, cytokines are a critical aspect of autocrine, paracrine, and endocrine signaling. Upon cytokine binding, some surface receptors activate the Janus kinase–signal transducer and activator of transcription (JAK-STAT) signaling pathway to regulate gene transcription (*1*). Alternatively, cytokine receptors cross-talk with other signaling macrodomains at the plasma membrane to regulate their posttranslational modifications (e.g., phosphorylation and ubiquitination) and activate additional signaling pathways [e.g., phosphatidylinositol 3-kinase (PI 3-kinase), nuclear factor κB (NF-κB), and mitogen-activated protein kinase (MAPK)] (*2*).

In addition to this extracellular role, many cytokines can also be found in the nucleus, and a functional role for the nuclear cytokine has been established in some cases (*3*–*7*). Conversely, the chromatin-associated protein HMGB1 is released from chromatin in dying cells, whereupon it acts in a cytokine-like manner (*8*). For two cytokines, chromatin immunoprecipitation (ChIP) experiments on a single locus suggest that they can associate with a specific genomic region (*5*, *6*). However, cytokine association with genomic regions has not been investigated on a genome-wide scale, and hence, little is known about how it might be recruited and how it affects transcription and biological phenotype.

Proinflammatory cytokines S100A8 and S100A9 (11 and 14 kDa, respectively) are calcium-binding proteins that form homodimeric, heterodimeric, and higher-order complexes that are essential for their biological activities (*9*–*11*). Up-regulation of S100A8/A9 occurs in various human cancer types including breast cancer, and both proteins are actively involved in tumor growth, metastasis, angiogenesis, and immune evasion in mouse models (*12*). Within the tumor microenvironment of breast cancer, S100A8 and S100A9 have both autocrine and paracrine functions. Extracellular S100A8/A9 released by cancer cells interacts with surface receptors including Toll-like receptor 4 (TLR4) and RAGE (Receptor for Advanced Glycation End products) (*13*, *14*), thereby promoting directed chemotaxis of myeloid cells and inducing proinflammatory responses and/or immune suppression by activation of JAK-STAT, NF-κB, and MAPK pathways (*12*, *15*, *16*). S100A8 and S100A9 are also abundant in the cytosol, where they help control cytoskeleton rearrangement and activate pathways involved in reactive oxygen or nitrogen species (*15*, *17*). In a mouse model of psoriasis, S100A9 was reported in the nucleus with a direct contribution to expression of the *C3* gene (*5*). However, the roles of S100A8/A9 and other cytokines in the nucleus are poorly understood.

Here, we show that S100A8/A9 is recruited to numerous promoters and enhancers in a model of breast cellular transformation. This recruitment is associated with multiple DNA sequence motifs recognized by DNA binding transcription factors, and it is linked to transcriptional activation. Nuclear-specific expression of S100A8/A9 increases oncogenic transcription and leads to enhanced breast transformation phenotype. These results suggest that, in addition to its classical cytokine functions, S100A8/A9 can act as a transcriptional coactivator.

## RESULTS

### S100A8/A9 is important for breast cellular transformation, and its overexpression is associated with certain forms of breast cancer

**Fig. 1:**
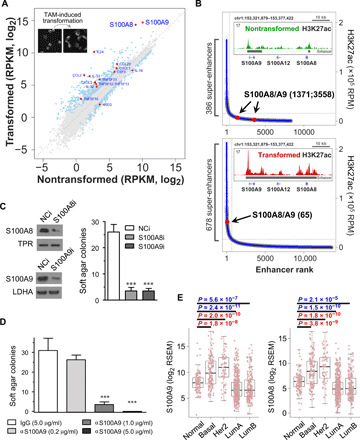
S100A8/A9 is important for breast cellular transformation. (**A**) Ribosome-associated RNA levels in nontransformed [ethanol (EtOH)–treated] and transformed [tamoxifen (TAM)–treated] cells. Differentially expressed genes are colored blue; cytokine genes that are differentially expressed are colored red. The inset shows morphology of cells during TAM-induced transformation. (**B**) Super-enhancer identification in nontransformed (top) and transformed (bottom) cells, with ranks of enhancers associated with S100A8/A9 shown in the parentheses. Insets show H3K27ac chromatin immunoprecipitation–sequencing (ChIP-seq) signal at the S100A8/A9 genomic region with enhancer or super-enhancer regions marked. (**C**) Western blot (left) and soft agar assay (right) with transformed cells transfected with negative control (NCi), S100A8, or S100A9 siRNA. Data are means ± SEM; *n* = 6; ***P < 0.001 compared with negative control treatment; *t* test. TPR, Translocated Promoter Region, Nuclear Basket Region. (**D**) Soft agar assay with cells induced by TAM together with indicated antibody treatment. Data are means ± SEM; *n* = 4; ***P < 0.001 compared with immunoglobulin G (IgG) treatment; *t* test. (**E**) RNA expression of S100A8 and S100A9 in PAM50-defined molecular subtypes of breast cancer. Wilcoxon rank sum test *P* values are shown. A total of 950 clinical breast samples from 840 patients in The Cancer Genome Atlas database were used in this analysis. RPKM, reads per kilobase of transcript per million mapped reads; RPM, reads per million; LDHA, lactate dehydrogenase A; RSEM, RNA-Seq by Expectation-Maximization.

Our inducible model of breast cellular transformation is based on a derivative of the nontransformed cell line MCF-10A that expresses ER-Src, a fusion between the v-Src oncoprotein and the ligand-binding domain of estrogen receptor (*18*, *19*). Treatment of these cells with tamoxifen activates v-Src and triggers an epigenetic switch between nontransformed and transformed cells that is mediated by inflammatory regulatory network controlled by the joint action of NF-κB, STAT3, and activating protein 1 (AP-1) transcription factors (*19*–*22*). On the basis of ribosome profiling experiments (*23*), many cytokine genes are induced upon transformation, with S100A8/A9 being near the most dominant and most up-regulated (Fig. 1A). H3K27 acetylation (H3K27ac) profiles (*21*) indicate that the S100A8/A9 gene locus is associated with a transformation-regulated super-enhancer (Fig. 1B), a cluster of enhancers that activate genes defining cell identity (*24*, *25*). Small interfering RNA (siRNA)–mediated knockdown (Fig. 1C) or antibody-mediated inhibition (Fig. 1D) of S100A8 and/or S100A9 efficiently blocks colony formation in soft agar, indicating that these cytokines are critical for transformation in our model. Last, transcriptional analysis of PAM50 (Prediction Analysis of Microarray 50)–defined molecular subtypes (*26*) of clinical breast cancer tissues from 840 patients (*27*) indicates that, compared with normal tissue, S100A8/A9 is overexpressed in basal-like and Her2-enriched subtypes that have poor clinical outcomes and underexpressed in the two luminal subtypes (Fig. 1E).

### Nuclear-localized S100A8/A9 increases transformation efficiency

**Fig. 2:**
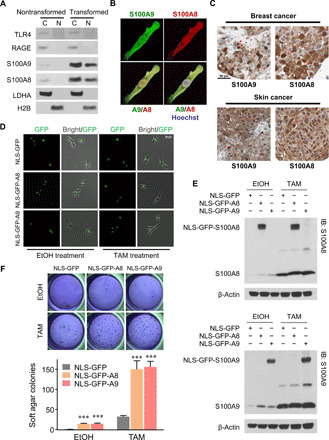
Nuclear S100A8/A9 and breast transformation. (**A**) Western blots of cytosolic (C) and nuclear (N) fractions in nontransformed and transformed cells. LDHA and histone 2B (H2B) are used as subcellular compartment markers. (**B**) Confocal imaging of S100A8 and S100A9 immunofluorescence costaining in a transformed cell. (**C**) S100A8 and S100A9 immunohistology from clinical samples of breast cancer (top) and skin cancer (bottom). Representative nuclear localizations of S100A8 or S100A9 are marked by arrows. Scale bar, 50 μm. Data are obtained from The Human Protein Atlas database. (**D**) Bright-field and GFP fluorescence imaging demonstrate nuclear-specific expression of the indicated derivatives in nontransformed and transformed cells. Scale bar, 50 μm. (**E**) Western blots of whole-cell lysates show expression of nuclear localization signal (NLS)–GFP–S100A8 (top) and NLS-GFP-S100A9 (bottom). IB, immunoblot. (**F**) Representative colony formation (top) and quantification (bottom) of soft agar assay with EtOH- or TAM-treated cells expressing indicated protein. Data are means ± SEM; *n* = 6; ***P < 0.001 compared with corresponding NLS-GFP group; *t* test. IB, Immunoblot.

Although S100A8 and S100A9 are secreted cytokines, cell fractionation experiments show that they are also observed in the nucleus, unlike their cognate receptors TLR4 and RAGE (Fig. 2A), suggesting that nuclear S100A8/A9 might have a function that is independent of the surface receptors. Immunofluorescence imaging of endogenous S100A8 and S100A9 with confocal microscopy shows well-overlapping signals, suggesting colocalization in the nucleus (Fig. 2B). Nuclear localization of S100A8/A9 is also observed in breast and skin cancer tissues from patients (Fig. 2C), thereby providing clinical relevance of the observation in the ER-Src cellular model.

To examine the function of nuclear S100A8/A9 in transformation, we constitutively expressed derivatives of these proteins that contain a nuclear localization sequence and green fluorescent protein (GFP) (for visualization). These hybrid proteins localize to the nucleus (Fig. 2D), and they are expressed at levels comparable to those of the endogenous proteins upon tamoxifen induction (Fig. 2E). Nuclear expression of S100A8 or S100A9 leads to a subtle transformation phenotype even in the absence of tamoxifen, and it enhances transformation efficiency in the presence of tamoxifen (Fig. 2F). These results indicate that nuclear S100A8/A9 plays a role in breast cell transformation.

### S100A8 and S100A9 target sites in the human genome

**Fig. 3:**
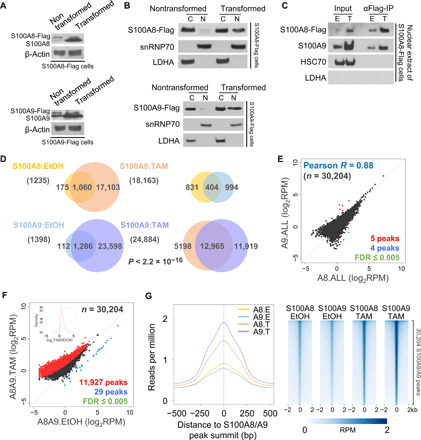
Genome-wide chromatin binding of S100A8 and S100A9. (**A**) Western blots of the indicated endogenous and ectopic proteins in whole-cell lysates of EtOH- or TAM-treated cells. (**B**) Western blots of Flag-tagged S100A8 (top) or S100A9 (bottom) in cytosolic and nuclear fraction under EtOH or TAM treatment. LDHA and snRNP70 are used as subcellular compartment markers. snRNP70, Small Nuclear Ribonucleoprotein U1 subunit 70. (**C**) Coimmunoprecipitation of Flag-tagged S100A8 with endogenous S100A9 in nuclear extract under EtOH or TAM treatment. HSC70 (Heat Shock Cognate 71 kDa protein) is used as a negative control; LDHA is used as a marker for the cytosolic compartment. (**D**) Venn diagrams show peak overlap of S100A8 and S100A9 binding in EtOH or TAM treatment. *P* < 2.2 × 10^−16^ for any of the peak overlap enrichment; Fisher’s exact test. (**E**) Relationship of S100A8 and S100A9 binding levels for the union of all peak regions of S100A8 or S100A9. (**F**) Combined S100A8 and S100A9 binding levels EtOH or TAM conditions. Peaks with statistically significant up- or down-regulation are colored red or blue, respectively. The inset shows the distribution of fold enrichment values for all S100A8/A9 peaks. (**G**) Binding profiles (average, left) and heatmaps (right) for S100A8 or S100A9 in EtOH- or TAM-treated condition around the summits of the union of all peak regions for S100A8 or S100A9. Replicate-pooled binding data are shown. Heatmaps are sorted by S100A9 ChIP signal in TAM treatment. FDR, false discovery rate; bp, base pairs.

To investigate the role of nuclear S100A8/A9, we performed ChIP-sequencing (ChIP-seq) using stable cell lines expressing Flag-tagged proteins at comparable levels of endogenous proteins in the absence of tamoxifen (Fig. 3A). The nuclear levels of the Flag-tagged proteins are strongly induced during transformation (Fig. 3B). We suspect that this apparent regulation of nuclear translocation of the Flag-tagged proteins reflects interactions with endogenous S100A8/A9, which is highly induced during transformation. In this regard, in nuclear extracts, S100A8-Flag interacts with endogenous S100A9 in a manner that is increased upon transformation increased upon transformation (Fig. 3C).

As S100A8 and S100A9 lack conventional DNA binding domains and hence are unlikely to bind DNA on their own, we used a dual–cross-linking approach that involves protein-protein cross-linking followed by traditional formaldehyde-mediated protein-DNA cross-linking (*28*, *29*). We performed ChIP-seq analysis in cells expressing Flag-tagged S100A8 or S100A9 in nontransformed and transformed conditions and obtained highly reproducible binding profiles (fig. S1). As expected from the increased nuclear protein levels upon tamoxifen induction, the number of binding sites (Fig. 3D) increases markedly during transformation, with nearly all the sites in nontransformed condition being observed during transformation. By combining S100A8 and S100A9 ChIP signals, we identified ~30,000 target sites, with extremely high correlation (*R* = 0.88) between S100A8 and S100A9 binding levels (Fig. 3E). Binding levels at essentially all sites are increased upon transformation, and the peak summits are coincident (Fig. 3, F and G). Thus, S100A8 and S100A9 specifically associate with common genomic regions, presumably by being recruited by specific DNA binding proteins. These results do not distinguish between homomeric S100A8 or S100A9 complexes and heteromeric S100A8/A9 complexes.

### S100A8/A9 binding sites are associated with active chromatin regions and polymerase II transcription

**Fig. 4:**
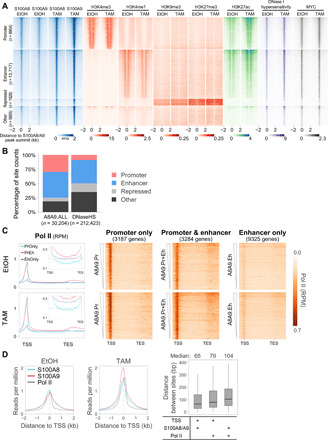
Epigenomic features of S100A8 and S100A9 chromatin binding. (**A**) Chromatin occupancy of indicated factors at ±2000 bp of summits of the union of all peak regions for S100A8 or S100A9 in combination with EtOH- and TAM-treated conditions. Heatmaps are segmented according to types of genomic regions as defined by patterns of histone modifications. Replicate-pooled chromatin binding data are shown for S100A8 and S100A9. Heatmaps are sorted by S100A9 ChIP signal in TAM treatment. (**B**) Percentage of the indicated classes of regions for all S100A8/A9 sites (A8A9.ALL) and DNase I hypersensitive sites (DNaseHS). (**C**) Meta-gene profiles (average, left) and heatmaps (right) of Pol II association from 1 kb upstream of transcription start site (TSS) and 2 kb downstream of transcription end site (TES) at indicated groups of S100A8/A9 target genes in EtOH and TAM treatment. Insets show Pol II signal at coding region; dash lines are aligned to 0.15. Heatmaps are sorted by S100A8/A9 ChIP signal density (±250 bp of peak summit) at sites at promoters (Pr), enhancers (Eh), or both (Pr + Eh). (**D**) Location of S100A8, S100A9, and Pol II signals with respect to the TSS along with box plots indicating the distance between pairwise combinations.

We classified S100A8/A9 peaks with respect to different types of regulatory regions as defined by histone modifications patterns (*21*, *30*). Specifically, 30% are located in promoter regions, 45% in enhancer regions, 6% in repressed regions, and 19% map to otherwise uncharacterized regions (Fig. 4A). When compared with the 212,423 deoxyribonuclease I (DNase I) hypersensitive sites identified in this transformation model (*21*), S100A8/A9 sites are strongly overrepresented in promoter and enhancer regions but depleted in repressed regions (*P* < 2.2 × 10^−16^, Fisher’s exact test; Fig. 4B). Furthermore, the level of S100A8/A9 binding is positively correlated with the levels of DNase I hypersensitivity (accessible chromatin), H3K27ac (active histone mark), and the transcriptional amplifier MYC (Fig. 4B and fig. S2A) at both promoter and enhancer regions, and the correlations increase mildly upon transformation. In contrast, the level of S100A8/A9 binding at repressed genomic regions is not or negatively correlated with the level of repressive histone modifications H3K9me3 and H3K27me3 (fig. S2B). These observations indicate that S100A8/A9 binding is associated with transcriptional activation.

To address the role of S100A8/A9 in transcription, we assigned peaks to neighboring genes and then subdivided them into genes with peaks only at promoters, only at enhancers, and at both promoters and enhancers (fig. S3A). Polymerase II (Pol II) occupancy at all these three groups of genes is higher in the transformed condition as compared with the nontransformed condition (Fig. 4C). Genes with S100A8/A9 sites in both promoter and enhancer regions show the strongest Pol II signals, particularly at coding regions, indicative of increased transcription (Fig. 4C). In contrast, genes with S100A8/A9 sites in enhancers only have considerably lower Pol II occupancy levels. In addition, there is modest correlation (0.24 to 0.34) between S100A8/A9 binding and Pol II occupancy at coding regions for all three categories and at the promoter, and minimal correlation to the pausing index (fig. S3B). Last, S100A8/A9 binding sites are located just upstream of the transcriptional start site and paused Pol II (Fig. 4D). These observations strongly suggest that nuclear S100A8/A9 is a transcriptional coactivator.

### Relationship of S100A8/A9 binding to gene induction during transformation and to human cancer genes

**Fig. 5:**
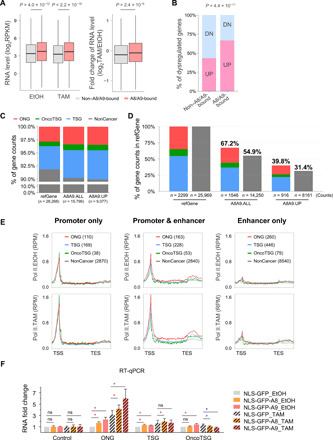
S100A8/A9 binding and cancer gene transcription. (**A**) Box plots on the left show expression levels of genes associated with (coral; 7679 genes) or without (gray; 2627 genes) S100A8/A9 binding sites in EtOH- or TAM-treated cells. Box plot on the right shows the fold change (TAM:EtOH) of the same classes of genes. Higher expression levels in both conditions and higher fold induction during transformation are associated with S100A8/A9-bound sites (Wilcoxon rank sum test *P* values indicated). (**B**) Percentage of genes differentially expressed (at least twofold) during transformation that are up-regulated (UP; pink) or down-regulated (DN; blue) and are associated with sites that are bound or unbound by S100A8/A9. Fisher’s exact test *P* value is indicated for the distribution comparison. (**C**) Gene counts and percentage of noncancer genes (NonCancer), oncogenes (ONG), TSGs, and genes annotated as both oncogenes and TSGs (OncoTSG) in hg19 refGene collection (refGene), all S100A8/A9 target genes (A8A9.ALL), and genes with strongly up-regulated S100A8/A9 peaks in TAM treatment (A8A9.UP). (**D**) Gene counts and percentage of cancer (left) and noncancer (right) genes from indicated gene collections. (**E**) Meta-gene average profiles of Pol II binding from 1 kb upstream of TSS and 2 kb downstream of TES at indicated groups of S100A8/A9 target genes in EtOH- and TAM-treated cells. Gene counts are shown in the parentheses. (**F**) Induction ratio (fold change relative to the mRNA level in EtOH-treated cells expressing NLS-GFP) of the indicated classes of genes (average of six cancer genes/class or two control genes) in cells expressing the indicated proteins. Data are means ± SEM; ns, not significant; *P < 0.05 compared with corresponding NLS-GFP group; Wilcoxon signed rank test. Asterisks (*) for increase were colored red or otherwise colored blue. RT-qPCR, reverse transcription quantitative polymerase chain reaction.

We analyzed the relationship between S100A8/A9 binding and gene expression upon transformation by comparing RNA expression levels of genes whose regulatory regions are bound by S100A8/A9 with those that are unbound. In both nontransformed and transformed conditions, the S100A8/A9 bound genes are expressed at higher levels than the unbound genes (Fig. 5A). In addition, the fold induction upon transformation is higher in S100A8/A9-bound genes than in the unbound genes (Fig. 5A). For genes differentially regulated upon transformation, 67% are up-regulated for the S100A8/A9 bound genes, whereas only 43% are up-regulated for the unbound genes (*P* = 4.4 × 10^−11^ by Fisher’s exact test; Fig. 5B). Thus, S100A8/A9 binding is preferentially associated with induction of genes during transformation.

S100A8/A9-bound target genes are enriched in functions linked to signaling (phosphorylation, acetylation, and glycoprotein), protein binding, mRNA processing, cytokine secretion, cell adhesion, and cell cycle (fig. S4). In addition, we addressed the relationship between S100A8/A9 target genes and human cancer gene annotations from UniProt (*31*), Network of Cancer Genes (*32*), Tumor Suppressor Gene database (*33*), ONGene (*34*), and Tumor Associated Gene database (*35*). We collected 808 oncogenes (ONGs), 1247 tumor suppressor genes (TSGs), and 244 cancer genes annotated as both ONGs and tumor suppressors (OncoTSGs). Among 15,796 S100A8/A9 target genes, 10% are human cancer genes with 533 ONGs, 843 TSGs, and 170 OncoTSGs (Fig. 5, C and D). Notably, 67% of annotated cancer genes are associated with S100A8/A9 target genes, in comparison to only 55% of noncancer genes, which is highly significant (*P* < 2.2 × 10^−16^; Fisher’s exact test) with contributions from ONG (*P* = 2 × 10^−8^), TSG (*P* = 2 × 10^−14^), and OncoTSG enrichment (*P* = 6 × 10^−6^). The distinction between cancer and noncancer genes is also observed when considering S100A8/A9 target sites showing the highest level of inducible binding upon transformation (Fig. 5, C and D). At target genes where S100A8/A9 binds at both the enhancer and promoter, ONGs have the higher level of transcriptional activity than other classes of genes (Fig. 5E).

To examine the function of nuclear S100A8/A9, we measured the expression of ONG, TSG, and OncoTSG genes (six genes for each class), whose promoters and enhancers are bound by S100A8/A9 in cells expressing S100A8 or S100A9 derivatives with a nuclear localization signal (NLS) (fig. S5). When considered as a class, the ONGs show increased expression upon ectopic expression of either S100A8 or S100A9, either in the presence or absence of tamoxifen (Fig. 5F). In contrast, this effect is smaller or not detectable on the TSGs and not detected on the OncoTSGs or control genes. Together, these observations preferentially link S100A8/A9 binding with expression of ONGs.

### Motif enrichment of transcription factors and chromatin association of S100A8/A9

**Fig. 6:**
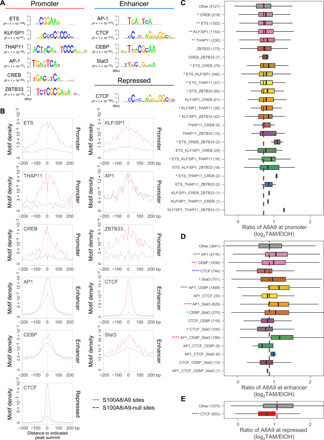
Motif enrichment and S100A8/A9 chromatin binding. (**A**) De novo identified DNA motifs at S100A8/A9 sites at the indicated classes of genomic regions highly matched to known motifs. The de novo motif search was performed for S100A8/A9 sites against DNase I hypersensitive sites lacking S100A8/A9 association (S100A8/A9-null sites) in the same class of chromatin regions. Hypergeometric *P* values for motif enrichments are shown. (**B**) Distributions of de novo discovered motif density at ±200 bp of the indicated peak summits of indicated classes of chromatin regions. (**C**) TAM-induced fold changes of S100A8/A9 chromatin binding at S100A8/A9 sites with or without indicated motifs (alone or in combination) in promoter regions. *P < 0.05, **P < 1 × 10^−5^, ***P < 1 × 10^−10^, and ****P < 2.2 × 10^−16^ compared with S100A8/A9 sites without indicated motifs (other); Wilcoxon rank sum test. Asterisks (*) for increased fold change were colored red or otherwise colored blue. Peak numbers of indicated groups are shown in the parentheses. The dashed line is aligned to median value of the Other group. (**D**) Same as (C) for sites at enhancer regions. (**E**) Same as (C) for sites at repressed regions.

As S100A8/A9 does not have intrinsic DNA binding activity, they are presumably recruited to genomic sites by association with specific DNA binding transcription factors. To identify these transcription factors, we performed de novo motif searches in S100A8/A9 binding sites in promoter, enhancer, and repressed regions. We identified ETS (Erythroblast Transformation Specific), KLF (Kruppel-Like Factor)/SP1 (Specificity Protein 1), THAP11 (THAP domain Containing 11), AP-1, CREB (cyclic adenosine monophosphate response element–binding protein), and ZBTB33 (Zinc finger and BTB domain containing 33), motifs enriched in promoter peaks; AP-1, CTCF (CCCTC-binding Factor), CEBP (CCAAT/Enhancer-Binding Protein), and STAT3 motifs enriched in enhancer peaks; and CTCF motif enriched in repressed peaks (Fig. 6A). Using sequence motifs for these factors, we then compared the probability of motifs in the set of S100A8/A9 binding regions with control regions from promoter, enhancer, and repressed DNase I hypersensitive sites lacking S100A8/A9 association (S100A8/A9-null sites; Fig. 6B). All these motifs are strongly enriched at S100A8/A9-bound regions as compared with control regions, except for the AP-1 motif at promoters that is only weakly enriched. Furthermore, the maximal motif densities map closely to the S100A8/A9 peak summits (Fig. 6B and fig. S6), consistent with the idea that S100A8/A9 is recruited (directly or indirectly) to target sites by the transcription factor(s) recognizing the motif.

For each motif or selected combination of motifs located within 50 base pairs (bp) of the S100A8/A9 peak summits, we determined the fold change of S100A8/A9 binding at that site during transformation (tamoxifen:ethanol ratio). At promoters, fold increases in S100A8/A9 binding observed at sites with ETS, KLF/SP1, THAP11, CREB, or ZBTB33 motif alone or in combination are subtle, although statistically significant when compared against target sites lacking any of the listed motifs (Fig. 6C). However, at enhancer regions, fold increases of S100A8/A9 binding are more pronounced, particularly at motifs for transcriptional activators AP-1, CEBP, or STAT3, alone or in combination (Fig. 6D). In contrast, the motif for the CTCF insulator protein, alone or in combination with transcriptional activators in either enhancer or repressed peaks, is associated with reduced binding of S100A8/A9 during transformation or counteraction of the positive effects from the activator motifs (Fig. 6E). These results are in line with the transcriptional coactivator role of S100A8/A9, and the connection to AP-1 and STAT3 is noteworthy given that these proteins are critical for transformation in this cellular transformation model (*20*–*22*).

### S100A8/A9 interacts with transcription factors in nuclear extracts and activates transcription upon artificial recruitment to a target promoter

**Fig. 7:**
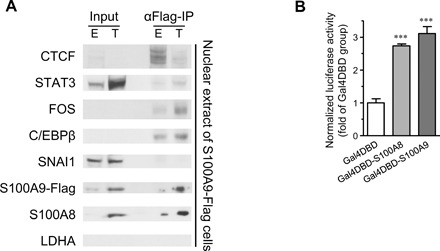
S100A8/A9 interacts with transcription factors and activates transcription upon artificial recruitment. (**A**) Western blot of S100A9-Flag immunoprecipitates of the indicated proteins in nuclear extracts from cells treated with EtOH (E) or TAM (T); lactate dehydrogenase (LDHA) is used as a cytoplasmic marker. (**B**) Transcriptional activity (normalized luciferase levels) in MCF7 cells electroporated with the indicated Gal4 DNA binding domain (DBD) protein derivative and the reporter construct containing five tandem repeats of the Gal4 binding site upstream of the core *Drosophila HSP70Bb* TATA-box promoter. Data are means ± SEM; *n* = 4; ***P < 0.001 compared with the Gal4DBD group; *t* test.

To examine more directly whether S100A8/A9 behaves like a coactivator, we first performed coimmunoprecipitation experiments in nuclear extracts from which nucleic acids were degraded. As expected from the motifs enriched among S100A8/A9 target sites, S100A9-Flag immunoprecipitates FOS, STAT3, C/EBPβ, and CTCF, but not SNAI1 (Fig. 7A). In accord with genomic binding levels (Fig. 6, D and E), the interactions of S100A9-Flag with FOS (and to a lesser extent, STAT3 and C/EBPβ) increases in transformed cells, whereas the interaction with CTCF decreases. Thus, S100A9 physically associates with multiple transcription factors, although these coimmunoprecipitation experiments do not address whether the interactions are direct or involve intermediary factors.

To examine whether S100A8/A9 has an autonomous transcriptional activation function, we fused the individual full-length proteins to the DNA binding domain of the yeast Gal4 proteins. When compared with the Gal4 DNA binding domain alone, the resulting fusion proteins confer about threefold higher levels of transcription from a Gal4-based reporter construct (Fig. 7B). These observations, together with the genomic binding profiles, indicate that S100A8/A9 acts as a transcriptional coactivator.

## DISCUSSION

### S100A8/A9 can act as a transcriptional coactivator

Cytokines are secreted molecules that bind and activate cell surface receptors, thereby transducing extracellular signals to effector proteins that regulate gene expression and other biological events. Many cytokines are also found in the nucleus and can be associated with chromatin, but the nuclear functions of cytokines are poorly understood. In two cases, S100A9 and interleukin-33, ChIP analysis of an individual gene has suggested that these cytokines could associate with a specific genomic region. Here, we show that S100A8/A9 associates specifically with numerous genomic regions even though the cognate receptors are absent from the nucleus. We provide evidence that S100A8/A9 functions as a transcriptional coactivator and show that nuclear-localized S100A8/A9 increases transformation efficiency.

Multiple lines of evidence support the conclusion that S100A8/A9 can act as a transcriptional coactivator. First, S100A8/A9 binding sites occur primarily in promoters and enhancers, and binding is associated with active chromatin regions and Pol II transcription. Second, most DNA sequence motifs associated with S100A8/A9 binding are recognized by transcriptional activator proteins, with the level of binding being increased at motifs of activators and decreased at motifs of CTCF, an insulator protein associated with repression. Third, in nuclear extracts, S100A9 coimmunoprecipitates and, hence, interacts (directly or indirectly) with proteins that recognize the motifs enriched among genomic sites bound by S100A8/A9. Fourth, nuclear-specific expression of S100A8/A9 increases transcription of many genes tested, with a preference for ONGs. Fifth, S100A8 and S100A9 can stimulate transcription from a reporter construct when artificially recruited by a heterologous DNA binding domain.

There are several possible mechanisms, not mutually exclusive, by which S100A8/A9 could function as a transcriptional coactivator upon recruitment to specific genomic sites. The artificial recruitment experiment (Fig. 7B) suggests that these proteins contain a transcriptional activation domain that directly stimulates transcription of the target gene. S100A8/A9 could also bind to a region of the recruiting protein that inhibits transcriptional activity, thereby increasing the activating potential of the recruiting protein. In a related manner, S100A8/A9 could cause a conformational change in the recruiting protein that increases its activation function. Last, these cytokines could mediate their effects through interactions with other recruited coactivators and/or other DNA binding transcription factors that functionally interact with factors bound to the enriched motifs. These potential mechanisms are not mutually exclusive.

### Nuclear S100A8/A9 is important for cellular transformation

S100A8/A9 is up-regulated and functionally important in many forms of human cancer (*12*, *36*, *37*), and it is required for transformation in our model of breast cellular transformation. It is generally assumed that the oncogenic roles of the S100A8/A9 cytokines reflect their function in extracellular signaling.

Our results strongly suggest that S100A8/A9 in the nucleus also plays a functional role in cellular transformation. First, S100A8/A9 target sites are preferentially associated with annotated cancer genes. Second, at target genes where S100A8/A9 binds at both the enhancer and promoter regions, ONGs have higher transcriptional activity than noncancer genes and other classes of cancer genes. Third, S100A8/A9 target sites are enriched for motifs of transcription factors important for transformation. AP-1 factors and STAT3 are particularly important for transformation in our model (*20*–*22*), and loss of ETS, KLF/SP1, THAP11, CREB, ZBTB33, or CEBP inhibits transformation or tumor growth in other cellular or animal models (*21*, *38*–*43*). Conversely, S100A8/A9 binding decreases at CTCF motifs during transformation, and CTCF haploinsufficiency in mice leads to increased cancer incidence (*44*). Fourth and most important, nuclear-specific expression of S100A8/A9 preferably promotes ONG transcription and enhances transformation efficiency. Although not directly proven, we suspect that the transcriptional coactivator function of S100A8/A9 provides the basis for how nuclear S100A8/A9 affects transformation.

Although the nuclear function of S100A8/A9 is important for transformation, antibodies against these proteins in the medium causes decreased transformation. These added antibodies are extracellular and unlikely to enter the nucleus, suggesting that the classic cytokine function of S100A8/A9 is also important for transformation. Thus, we suggest the S100A8/A9 plays a role in transformation both as a classic cytokine that stimulates signaling pathways and as a transcriptional coactivator in the nucleus. In this regard, S100A8/A9 resembles β-catenin, a dual-function protein that acts as transcriptional coactivator in the nucleus and as a subunit of the cadherin complex involved in cell-cell adhesion (*45*).

## MATERIALS AND METHODS

### Antibodies and primer pairs for quantitative polymerase chain reaction analysis

The information for these reagents is described in table S1.

### Cell culture

MCF-10A cells expressing ER-Src (MCF-10A–ER-Src cells) were grown in DMEM/F-12 (Dulbecco’s Modified Eagle Medium/Nutrient Mixture F-12) with 5% charcoal-stripped fetal bovine serum (Sigma-Aldrich) and supplements as previously described (*19*). To induce transformation, cells were treated with 1 μM 4-hydroxy-tamoxifen (Sigma-Aldrich) or ethanol as vehicle control for 24 hours. Human embryonic kidney (HEK) 293T and MCF7 cells were grown in DMEM with 10% fetal bovine serum (Sigma-Aldrich). For the soft agar assay, cells (2.5 × 10^4^) were washed with phosphate-buffered saline (PBS) and treated with trypsin. The trypsinized single cells were resuspended in the upper agar phase (growth medium containing final 0.4% agar, freshly prepared at 37°C), which was layered onto the lower agar phase (growth medium containing 1.0% agar). The solidified agar was covered with growth medium, which was changed every 3 days. Colonies (>0.01 mm^2^) were counted with ImageJ after 30 days.

### Cloning and expression of S100A8/A9 derivatives

For S100A8-Flag or S100A9-Flag, C-terminal 1× Flag-tagged S100A8 or S100A9 open reading frame was cloned from the cDNA library of MCF-10A–ER-Src cells and then were subcloned into pLHCX retroviral vector (Clontech, #631511). For NLS-GFP, GFP with triplicated NLS (sequence copied from Invitrogen’s pCMV/myc/nuc) at both the N- and C-terminal sides was cloned from pEGFP-N1 (provided by R. Pomerantz from Temple University School of Medicine) using ultramer primers and then was subcloned into pLHCX. For NLS-GFP-A8 or NLS-GFP-A9, S100A8 or S100A9 open reading frame was subcloned to the C-terminal side of NLS-GFP in pLHCX.

Retrovirus was produced by transfection of corresponding retroviral vector in HEK293T cells carrying pCL-Ampho (provided by V. Sukhatme). Retroviral supernatant from HEK293T cells was collected at 48 and 72 hours after transfection, filtered through 0.45-μm filter, and used to infect exponentially growing target cells in the presence of polybrene (8 μg/ml; Millipore). Cells were passaged after 72 hours of infection, after which drug selection was performed with hygromycin B (500 μg/ml; Santa Cruz Biotechnology). For nuclear-specific expression of S100A8 or S100A9, cells expressing NLS-GFP, NLS-GFP-A8, or NLS-GFP-A9 were further selected by a cell sorting flow cytometer (SH800Z, Sony) based on GFP signals, and GFP levels for all groups of cells were kept similar on average during the selection.

### Gal4-based luciferase reporter analysis

Gal4 DNA binding domain (DBD)–based constructs were generated by replacing the NLS-GFP region of NLS-GFP, NLS-GFP-S100A8, or NLS-GFP-S100A9 with Gal4DBD cloned from pCAG-GBP1-10gly-Gal4DBD (plasmid #49438, Addgene). For the Gal4-based reporter assays, Gal4-based constructs (1 μg), pUAS-luc2 (1 μg; plasmid #24343, Addgene), and pCMV-RL (100 ng; catalog #E2261, Promega) were electroporated together into human breast MCF7 cells by the 4D-Nucleofector System with the SE Cell Line 4D-Nucleofector X Kit (Lonza). Forty-eight hours after the electroporation, *Firefly* and *Renilla* luciferase activities were measured with the Dual-Glo Luciferase Assay System (Promega). Luciferase activity is calculated as *Firefly* normalized by *Renilla*.

### Protein analysis

Cells were lysed in 1× radioimmunoprecipitation assay (RIPA) buffer (Cell Signaling Technology) supplemented with protease inhibitor cocktail (Sigma-Aldrich), followed by brief sonication, and the lysate was cleared by centrifugation at 20,000*g* for 10 min. For cytosol/nuclear fractionation, cells were incubated in buffer A (10 mM Hepes-KOH at pH 7.5, 50 mM NaCl, 500 mM sucrose, 0.1 mM EDTA, 1.5 mM MgCl_2_, and protease inhibitor cocktail) for 15 min, followed by mixture with Triton X-100 (0.5% at final) for 1 min, and the cytosol fraction was cleared by centrifugation at 16,000*g* for 5 min. The nuclear pellet was briefly washed in buffer B (15 mM Hepes-KOH at pH 7.5, 60 mM KCl, 15 mM NaCl, 0.32 mM sucrose, and protease inhibitor cocktail), dissolved in 1× RIPA buffer, and briefly sonicated, and the nuclear fraction was cleared by centrifugation at 20,000*g* for 10 min.

For coimmunoprecipitation, the nuclear pellet from cells expressing S100A8-Flag was dissolved in buffer C (50 mM tris-HCl at pH 8.0, 420 mM NaCl, 1.5 mM MgCl_2_, 0.4% NP-40, 25% glycerol, and protease inhibitor cocktail) for 1 hour, diluted to 100 mM NaCl strength with buffer D (50 mM tris-HCl at pH 8.0, 0 mM NaCl, 1.5 mM MgCl_2_, 0.4% NP-40, 25% glycerol, and protease inhibitor cocktail), and treated overnight with Benzonase (Millipore), and the nuclear extract was cleared by centrifugation at 20,000*g* for 10 min. Coimmunoprecipitation and Western blot analyses were performed as previously described (*46*).

For imaging analysis, cells were fixed with 4% paraformaldehyde in PBS, and immunofluorescence staining was performed with rabbit anti-S100A9 and mouse anti-S100A8, followed by treatment with anti-rabbit immunoglobulin G (IgG)–fluorescein isothiocyanate and anti-mouse IgG–Texas Red. Clinical immunohistology data of S100A9 and S100A8 were obtained from The Human Protein Atlas database (*47*); for breast cancer, S100A9 staining data were from PatientID_2091, and S100A8 staining data were from PatientID_2392; for skin cancer, S100A9 staining data were from PatientID_2166, and S100A8 staining data were from PatientID_2150.

### RNA interference

siRNAs of negative control, S100A8 (HSC.RNAI.N002964.12.3), and S100A9 (HSC.RNAI.N002965.12.1) were obtained from Integrated DNA Technologies. siRNA-mediated knockdown was performed as previously described (*48*). After 3 days of siRNA transfection, cells were tested for tamoxifen-induced transformation.

### RNA analysis

RNA was extracted with QIAzol (QIAGEN) and precipitated with isopropanol. cDNA was generated from 3.5 μg of total RNA template using oligo(dT)_20_ and SuperScript III (Invitrogen). Analysis of relative RNA expression was performed by quantitative polymerase chain reaction in real time. The relative RNA levels of indicated genes were normalized to the average RNA level of two control genes rRNA18S and FAM50A in the same samples. Data presented were obtained from four independent experiments.

### Analysis of ribosome profiling and RNA sequencing data

Ribosome profiling and RNA sequencing (RNA-seq) experiments were described previously (*23*). For ribosome profiling, adapter sequence at the 3′ end and first nucleotide at the 5′ end of each FASTQ read were trimmed. Trimmed reads were mapped to human ribosomal (rRNA) reference sequences (NR_003285.2, NR_003286.1, NR_003287.1, and NR_023363.1) with Bowtie (*49*). rRNA-unmapped reads were aligned to hg19 refGene and genome sequence with TopHat (*50*). Only uniquely mapped reads were used for further analysis. Ribosome occupancy was used to measure gene expression and was calculated as reads per kilobase of transcript per million mapped reads (RPKM) in defined regions of gene open reading frames (ORFs), which excluded 15 codons downstream of start codons and 5 codons upstream stop codons as well as the coding regions that overlapped with upstream ORFs we annotated previously (*23*) to improve accuracy of the measurement. For RNA-seq, sequencing reads were mapped with the same procedure as described in ribosome profiling. RNA expression levels of genes were calculated as RPKM. For reliable measurement of RNA levels and fold changes, at least five raw reads for each transcript were required.

### Chromatin immunoprecipitation–sequencing

Cells expressing S100A8-Flag, S100A9-Flag, or untagged protein were grown in the presence or absence of tamoxifen, washed once with room temperature PBS, and then fixed with protein-protein cross-linking solution [1.5 mM ethylene glycol-bis (EGS), 1.5 mM disuccinimidyl glutarate (DSG), and 1.5 mM disuccinimidyl suberate (DSS) in PBS] for 45 min at room temperature. Then, protein-DNA cross-linking with 1% formaldehyde (methanol free) was performed for 15 min at room temperature, and the reaction was quenched by 125 mM glycine. For Pol II ChIP, standard formaldehyde cross-linking was performed. After fixation, nuclei were isolated and dissolved in sonication buffer (10 mM tris-HCl at pH 8.0, 100 mM NaCl, 1 mM EDTA, 0.5 mM EGTA, 0.1% sodium deoxycholate, 0.5% *N*-lauroylsarcosine, and protease inhibitor cocktail) for sonication and subsequent immunoprecipitation (Flag antibody was used for negative control or S100A8 or S100A9 ChIP; Pol II antibody, 8WG16, was used for Pol II ChIP). The sheared chromatin fragments were enriched around 200 to 300 bp. For ChIP and corresponding chromatin input samples, DNA was isolated and used to prepare libraries, which were sequenced on Illumina NextSeq 500 or HiSeq 2000. Other ChIP-seq (H3K4me3, H3K4me1, H3K9me3, H3K27me3, H3K27ac, and MYC) and DNase sequencing (DNase-seq) experiments mentioned in this study were performed in transformed or nontransformed cells as previously described (*21*, *51*).

### Analysis of ChIP-seq data

Sequencing FASTQ reads were aligned to the hg19 human reference genome with Bowtie2 (*52*) with the following restrictions “--very-sensitive --score-min L,-0.6,-0.15” for mapping sensitivity and accuracy. Only uniquely mapped reads, and in the case of paired-end sequenced data, only uniquely mapped sequence pairs that were aligned concordantly, were used for subsequent analyses. In S100A8 or S100A9 ChIP-seq data, confident peaks were defined as fulfilling all the following restrictions: (i) MACS2 peak-calling *q* value ≤1 × 10^−7^ against sample-corresponding chromatin input background; (ii) peaks called by MACS2 were identified in at least two biological replicates; (iii) at least five raw reads were aligned at the peak region. Depending on the comparisons indicated, peak regions from different datasets were merged if overlapping at least 1 bp. Confident Pol II ChIP-seq peaks were defined as MACS2 paired-end peak-calling *P* value ≤1 × 10^−8^ against genomic background of 1-Mb genomic region around. In ChIP-seq data of histone modifications and DNase-seq data, confident sites were defined as previously described (*21*). Meta-gene and heatmap analyses were carried out with deepTools (*53*).

### Gene target mapping of S100A8/A9 peaks

Gene annotation and information of transcription start sites (TSSs) in hg19 refGene collection were downloaded from UCSC (University of California Santa Cruz) Genome Browser. Classification of S100A8/A9 peaks was determined by patterns of histone modifications. For promoter-bound S100A8/A9 peaks, genes were assigned by mapping the nearest annotated TSS to the peak summit if the peak summit was within 1500 bp upstream and 250 bp downstream of the TSS. If the peak summit was located downstream of the TSS even though within the 250-bp range, we further required that the distance of the peak summit to TSS be <5% of the gene length because the promoter region of a given gene should be around TSS but not largely extended to gene body region. For enhancer-bound S100A8/A9 peaks, genes were assigned either if their TSSs were within 50 kb the of peak summit or if the gene regions overlapped with the peak summit.

### Pol II density and pausing index

Promoter proximal was defined as ±250 bp around TSS, and gene body was defined as the remaining length of gene. Calculation of Pol II density as reads per million per kilobase (RPM/kb) was carried out in promoter proximal and gene body of each gene longer than 251 bp. The Pol II density ratio of promoter proximal and gene body was defined as pausing index or traveling ratio (*54*, *55*).

### Super-enhancer analysis

H3K27ac ChIP-seq signal at enhancer peaks, where H3K4me3 was lacking, was used to identify super-enhancers by ROSE algorithm (*25*). H3K27ac enhancer peaks were stitched if they were within distance of 12.5 kb. Gene annotations were assigned to H3K27ac enhancer peaks (stitched or not, depending on the distance within 12.5 kb or not) if TSSs were within 50-kb distance of peak or if gene regions overlapped with peak.

### Motif discovery

De novo motifs were searched with HOMER (*56*) in sequences ±100 bp around summits of promoter, enhancer, or repressed S100A8/A9 peaks against corresponding control regions from promoter, enhancer, and repressed DNase I hypersensitive sites lacking S100A8/A9 association. The enriched motifs (according to hypergeometric *P* values) were compared with JASPAR CORE collection of vertebrate motifs (*57*) for scoring their similarity (by HOMER), which were less than or equal to 1 (complete similarity). Discovered motifs with similarity score >0.9 were selected for subsequent analyses.

### Collection of human cancer genes

We collected and consolidated annotations of human cancer genes from the following sources: (i) 420 human cancer genes from UniProt (*31*) with keyword “KW-0656” for ONGs and keyword “KW-0043” for tumor suppressors, (ii) 711 known cancer genes and tumor suppressor/ONG annotations from the Network of Cancer Genes (*32*), (iii) 1217 human TSGs from the Tumor Suppressor Gene database 2.0 (*33*), (iv) 803 human ONGs from the ONGene database (*34*), and (v) 239 human ONGs and 251 human tumor suppressors according to annotations from the Tumor Associated Gene database (*35*).

### The Cancer Genome Atlas data

RNA-seq gene expression data for 950 clinical breast samples from 840 patients were downloaded from The Cancer Genome Atlas (TCGA). Molecular subtypes of breast cancer were defined by PAM50 classification (*26*).

### Gene ontology analysis

Gene ontology enrichment was calculated in the Database for Annotation, Visualization, and Integrated Discovery (DAVID) online server with default knowledgebase mapping (*58*).

### Statistics

Statistical tests of differential chromatin binding were carried out with DESeq2 algorithm integrated in R package DiffBind (*59*). Calculation of Pearson correlation coefficients and statistical tests was carried out with R function cor.test. Enrichment of gene set or peak set was determined by Fisher’s exact test with R function fisher.test. Statistical significance otherwise was determined as indicated.

### Data availability

Sequencing data have been deposited in the National Cancer for Biotechnology Information Gene Expression Omnibus (GEO) and are accessible through GEO accession number GSE155421.

## Supplementary Material

http://advances.sciencemag.org/cgi/content/full/7/1/eabe5357/DC1

Adobe PDF - abe5357_SM.pdf

S100A8/S100A9 cytokine acts as a transcriptional coactivator during breast cellular transformation

## SUPPLEMENTARY MATERIALS

Supplementary material for this article is available at http://advances.sciencemag.org/cgi/content/full/7/1/eabe5357/DC1

## Appendix

View/request a protocol for this paper from *Bio-protocol*.
