# Supplementary material for: S100A8/S100A9 cytokine acts as a transcriptional coactivator during breast cellular transformation
Source: Sci Adv. 2021 Jan 1;7(1):eabe5357. doi: 10.1126/sciadv.abe5357 (PMC7775746; doi:10.1126/sciadv.abe5357)
Supplement: http://advances.sciencemag.org/cgi/content/full/7/1/eabe5357/DC1 [file supp_7_1_eabe5357__index.html]

Science Advances | Science AdvancesAAASSearchScience AdvancesMenu

## Supplementary Materials

# S100A8/S100A9 cytokine acts as a transcriptional coactivator during breast cellular transformation

Ruisheng Song, Kevin Struhl

Download Supplement

**This PDF file includes:**

- Figs. S1 to S6
- Table S1

**Files in this Data Supplement:**

- Adobe PDF - abe5357\_SM.pdf
