# Supplementary material for: S100A8/S100A9 cytokine acts as a transcriptional coactivator during breast cellular transformation
Source: Sci Adv. 2021 Jan 1;7(1):eabe5357. doi: 10.1126/sciadv.abe5357 (PMC7775746; doi:10.1126/sciadv.abe5357)
Supplement: http://advances.sciencemag.org/cgi/content/full/7/1/eabe5357/DC1 [file supp_7_1_eabe5357__1.pdf]

Science Advances
AAAS

advances.sciencemag.org/cgi/content/full/7/1/eabe5357/DC1

Supplementary Materials for

S100A8/S100A9 cytokine acts as a transcriptional coactivator during breast cellular transformation

Ruisheng Song and Kevin Struhl*

*Corresponding author. Email: kevin@hms.harvard.edu

Published 1 January 2021, *Sci. Adv.* **7**, eabe5357 (2021)
DOI: 10.1126/sciadv.abe5357

This PDF file includes:

Figs. S1 to S6
Table S1

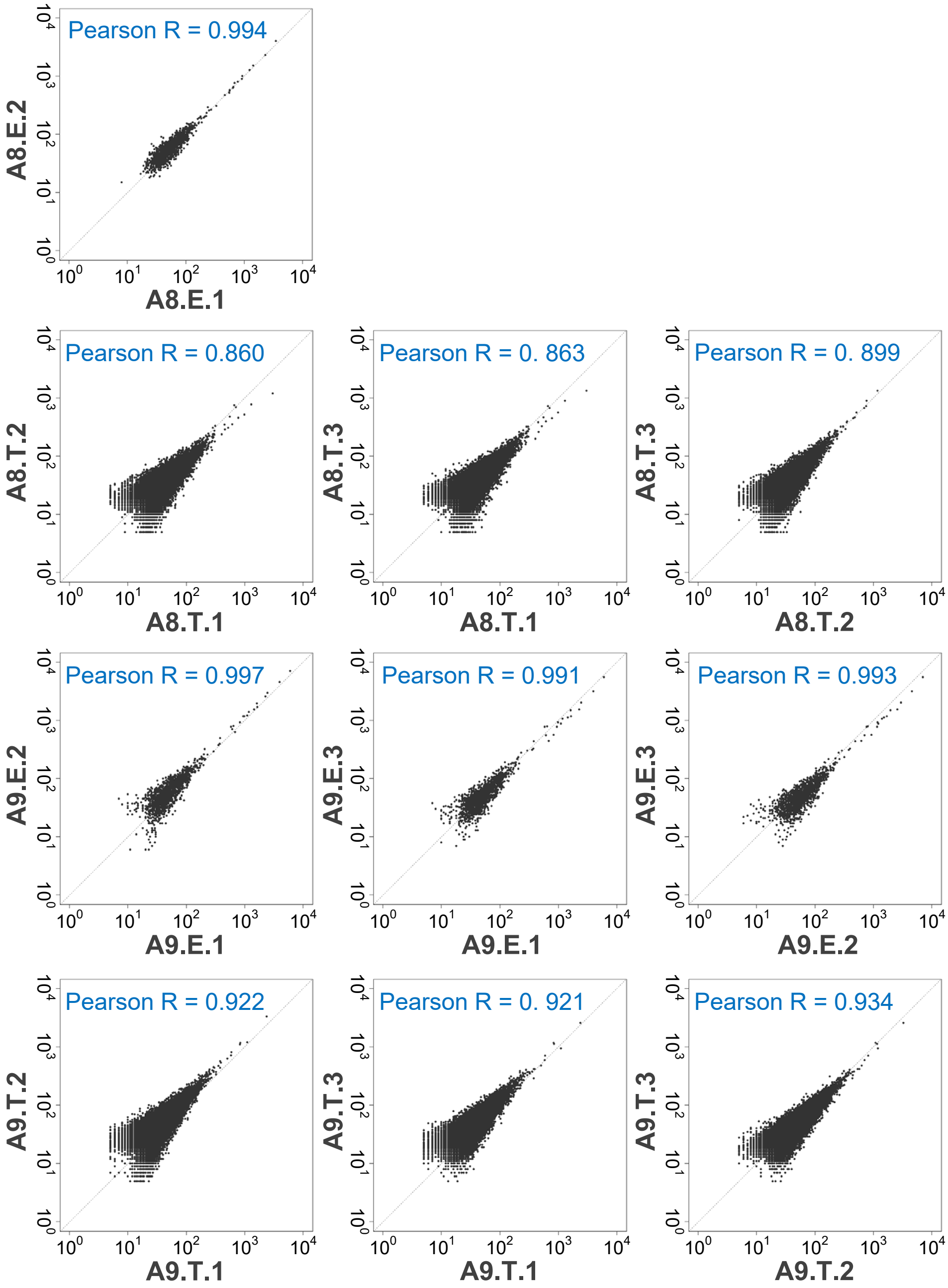

Fig. S1. **Genome-wide S100A8 and S100A9 chromatin binding.** Scatterplots show correlation of S100A8 or S100A9 biological replicates in EtOH or TAM treatment. Raw read counts at the union of all peak regions of indicated two samples in comparison are shown.

Fig. S1

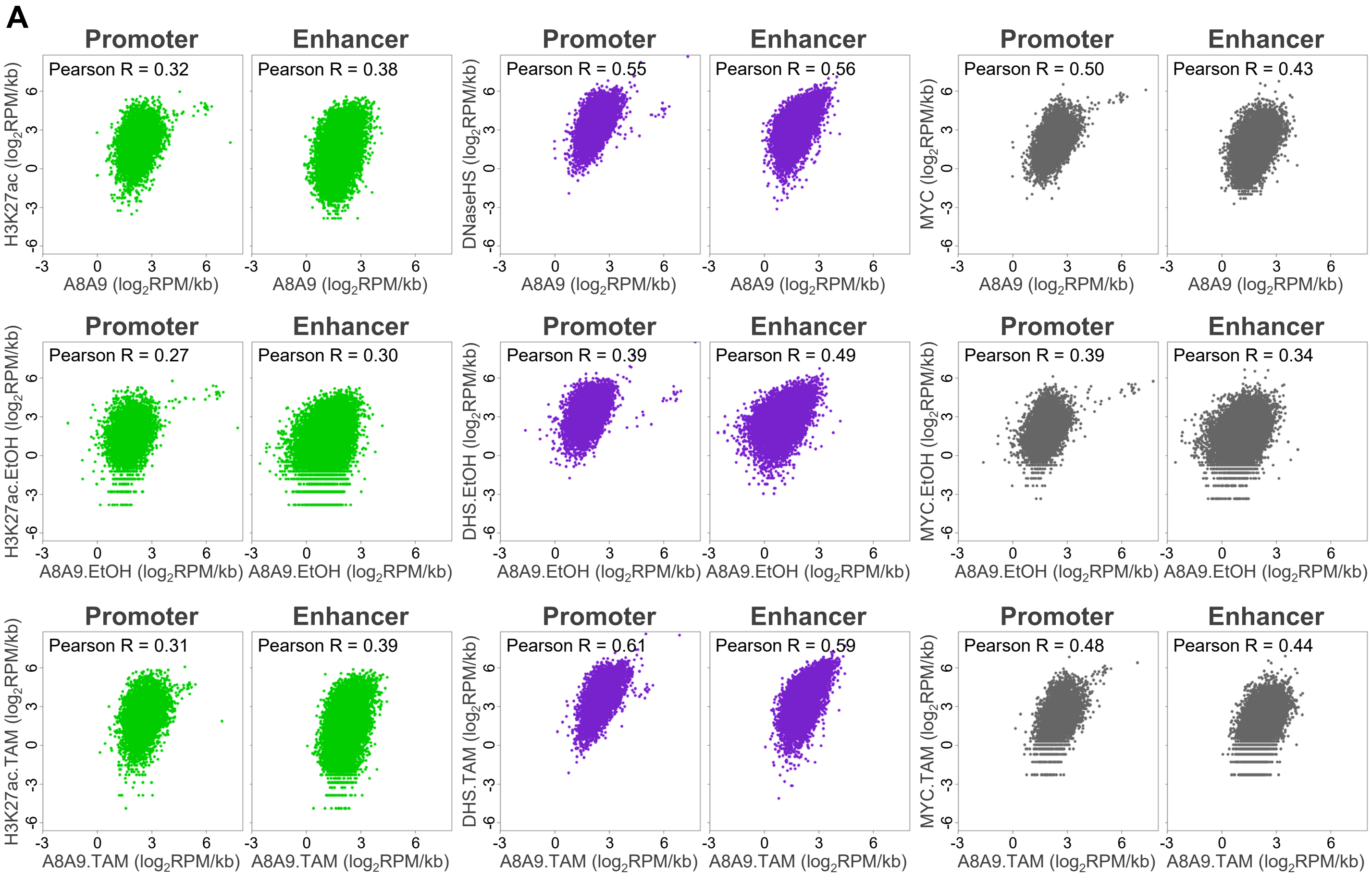

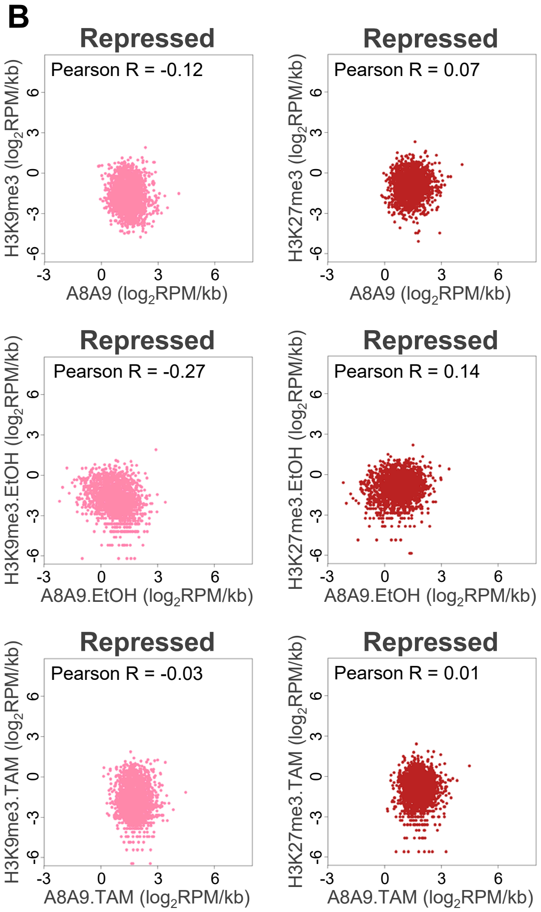

Fig. S2. Relationship of S100A8 and S100A9 binding to chromatin features. (**A**) Relationship of S100A8/A9 binding with H3K27ac (green), DNase I hypersensitivity (DNaseHS, purple) and MYC (grey) binding signals in EtOH- (middle) or TAM-treatment (lower) or in combination (upper) ±250 bp (S100A8/A9, DNaseHS and MYC) or ±1000 bp (H3K27ac) of summits of the union of all promoter or enhancer peak regions of S100A8 or S100A9. (**B**) Relationship of S100A8/A9 binding with H3K9me3 (pink) and H3-K27me3 (red) in EtOH- (middle) or TAM-treatment (lower) or in combination (upper) at ±250 bp (S100A8/A9, DNaseHS and MYC) or ±1000 bp (H3K9me3 and H3K27me3) of summits of the union of all repressed peak regions of S100A8 or S100A9.

Fig. S2

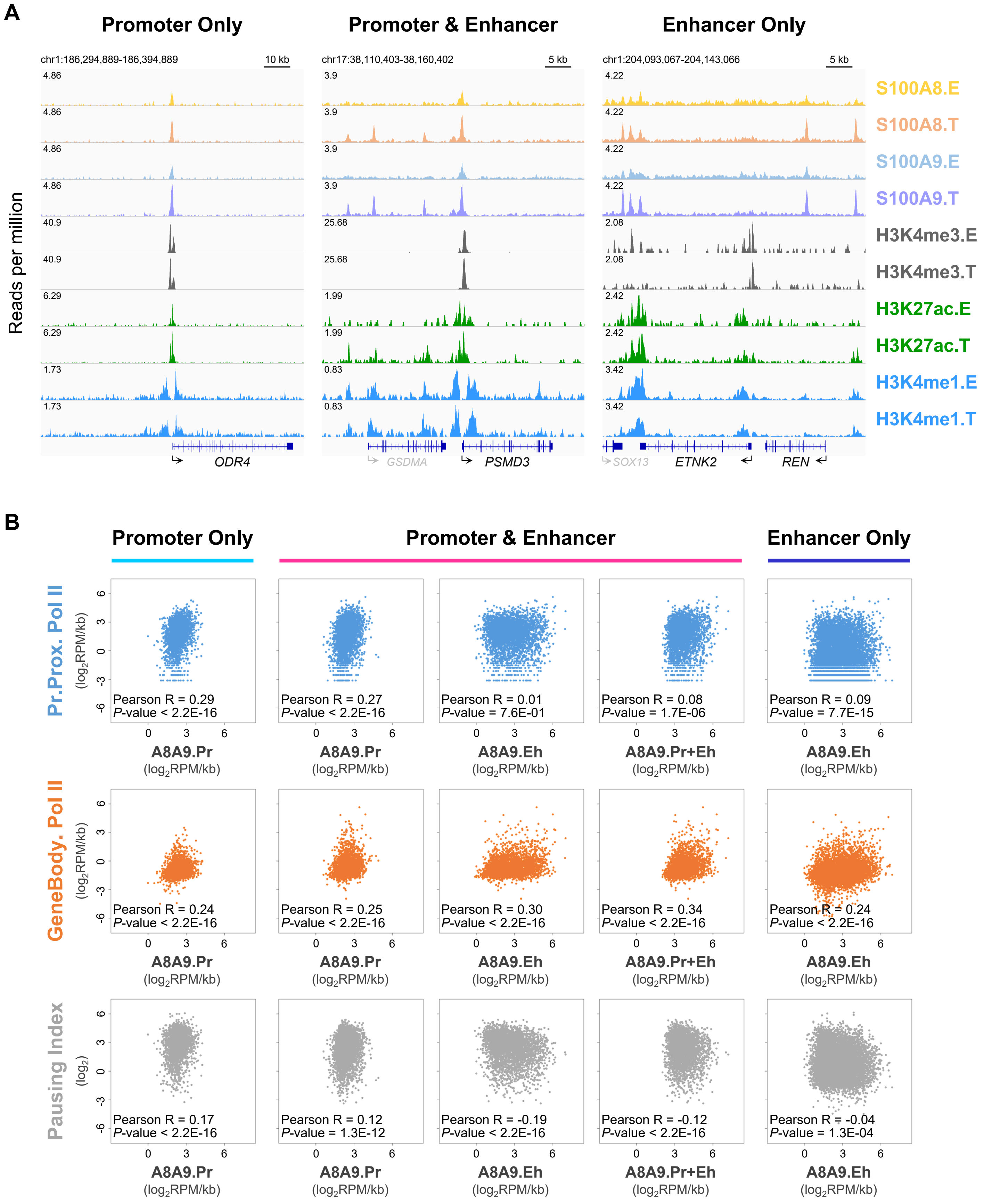

Fig. S3

Fig. S3. Classes of S100A8/A9 target genes and relationship of S100A8/A9 binding and Pol II transcription. (**A**) ChIP occupancies of indicated factors at indicated genomic loci in EtOH and TAM conditions, illustrating gene(s) with S100A8/A9 peak(s) only at promoter (left, *ODR4*), only at enhancers (right, *ETNK2* and *REN*; middle, *GSDMA*), and at both promoter and enhancers (middle, *PSMD3*). (**B**) Relationship of S100A8/A9 binding (±250 bp of peak summit) at promoter (Pr), enhancer (Eh) or both (Pr+Eh) as indicated with Pol II at promoter proximal (Pr.Prox, upper/blue) and gene body (GeneBody, middle/orange) locations as well as Pol II pausing index (lower/grey) at indicated groups of S100A8/A9-target genes.

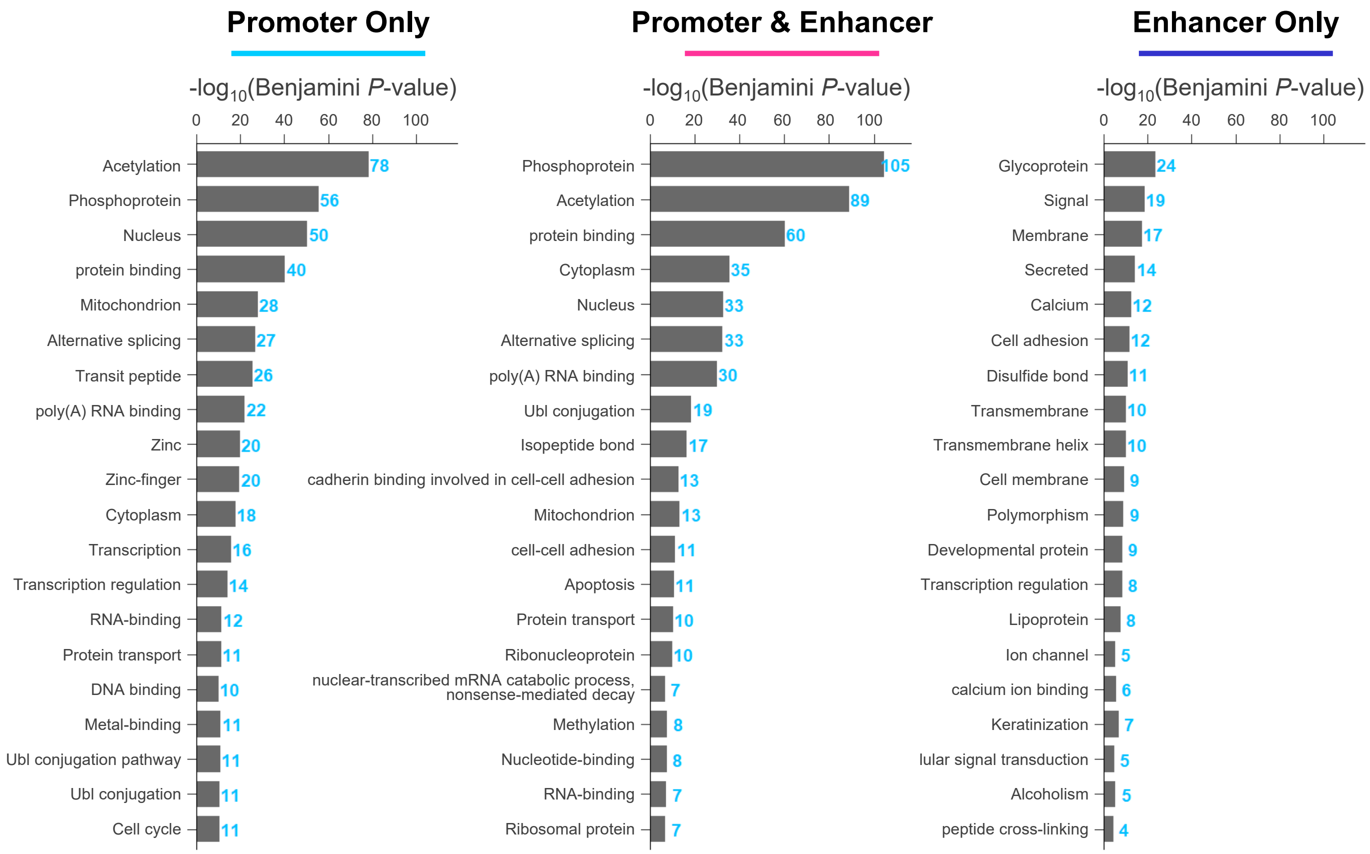

Fig. S4. **Gene ontology of S100A8/A9 target genes.** Top twenty enriched functional annotation terms are shown for genes where S100A8/A9 are bound at promoters only (left), enhancers only (right), or both promoters and enhancers (middle). Values of -log10(Benjamini *P*-value) are also labelled on top of the bars and colored blue.

Fig. S4

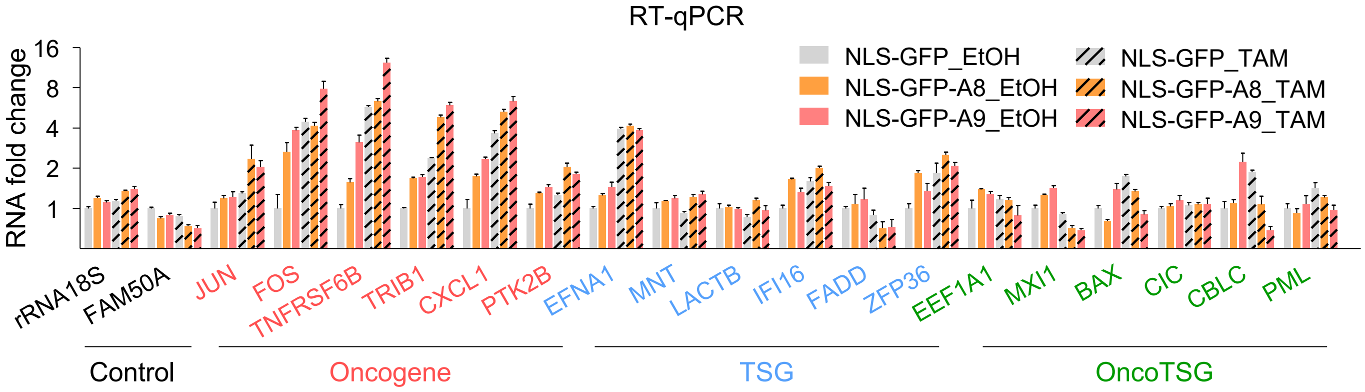

Fig. S5. **Transcription in cells expressing nuclear-localized S100A8/A9.** Induction ratio (RNA levels with respect to ethanol-treated cells expressing NLS-GFP whichis defined as 1) of the indicated classes of genes (average of 6 genes/class) in cells expressing the indicated proteins. Data are mean ± s.e.m; n = 3~4; *y*-axis is in log2 scale.

Fig. S5

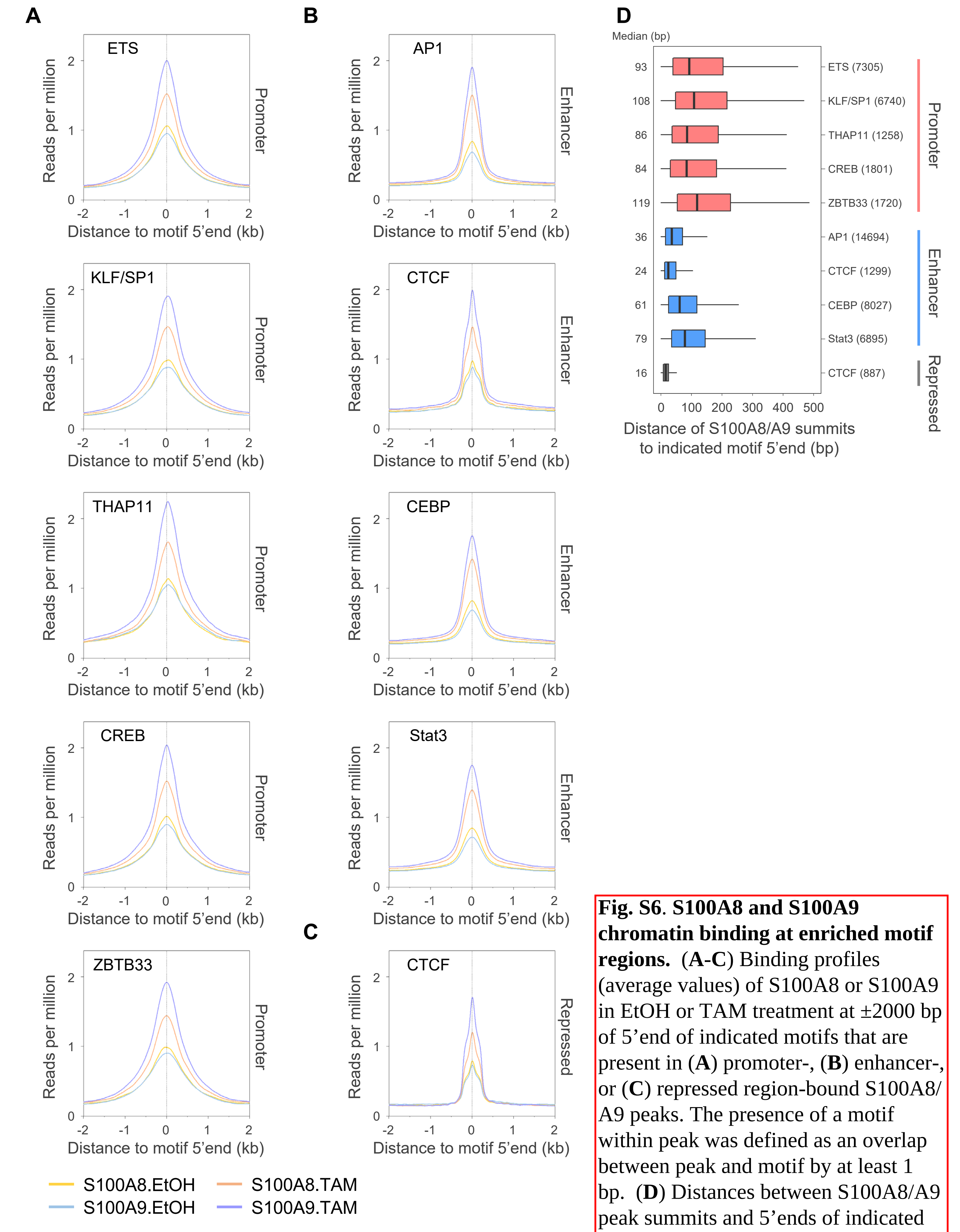

Fig. S6

Fig. S6. S100A8 and S100A9 chromatin binding at enriched motif regions. (**A-C**) Binding profiles (average values) of S100A8 or S100A9 in EtOH or TAM treatment at ±2000 bp of 5'end of indicated motifs that are present in (**A**) promoter-, (**B**) enhancer-, or (**C**) repressed region-bound S100A8/A9 peaks. The presence of a motif within peak was defined as an overlap between peak and motif by at least 1 bp. (**D**) Distances between S100A8/A9 peak summits and 5'ends of indicated motifs described in (**A-C**).

Antibodies and RT-qPCR Primers

Antibody	Catalog No.	Manufacturer
anti-S100A8	sc-48352	Santa Cruz Biotechnology
anti-S100A9	sc-20173	Santa Cruz Biotechnology
anti-H2B	sc-10808	Santa Cruz Biotechnology
anti-Flag epitope	2368	Cell Signaling Technology
anti-LDHA	2012	Cell Signaling Technology
anti-TLR4	AF1478	R&D Systems
anti-RAGE	AF1145	R&D Systems
anti-Pol II (8WG16)	MMS-126R	Covance
anti-TPR	A300-827A	Bethyl Laboratories
anti-CTCF	3418	Cell Signaling Technology
anti-FOS	2250	Cell Signaling Technology
anti-STAT3	12640	Cell Signaling Technology
anti-C/EBPβ	sc-7962	Santa Cruz Biotechnology
anti-SNAI1	3895	Cell Signaling Technology
anti-rabbit IgG-HRP	sc-2317	Santa Cruz Biotechnology
anti-mouse IgG-HRP	sc-2318	Santa Cruz Biotechnology
anti-mouse IgGϰBP-HRP	sc-516102	Santa Cruz Biotechnology
anti-goat IgG-HRP	sc-2033	Santa Cruz Biotechnology
anti-rabbit IgG-FITC	sc-2012	Santa Cruz Biotechnology
anti-mouse IgG-TexasRed	sc-2781	Santa Cruz Biotechnology

RT-qPCR Target Gene	Primer Sequence
rRNA18S	forward 5' GGAAGGGCACCACCAGGAGT 3'<br>reverse 5' TGCAGCCCCGGACATCTAAG 3'
FAM50A	forward 5' GCTTCTTGCCTGATCGAGACCGTGA 3'<br>reverse 5' TGCTCCACCCCTGCGGACCT 3'
JUN	forward 5' CTTTCCTGCGTCTTAGGCTT 3'<br>reverse 5' TCGCCCAAGTTCAACAACCG 3'
FOS	forward 5' CCAGTGCCAACTTCATTCCC 3'<br>reverse 5' TAGTTGGTCTGTCTCCGCTT 3'
TNFRSF6B	forward 5' CCGCTACTGCAACGTCCTCT 3'<br>reverse 5' CGATGACGGCACGCTCACAC 3'
TRIB1	forward 5' CCGGCTCTTCAAGCAGATTGTCTC 3'<br>reverse 5' GCAGAACTGTCCACGCCGAAT 3'
CXCL1	forward 5' AACATCCAAAGTGTGAACGTGA 3'<br>reverse 5' GAACAGCCACCAGTGAGCTTC 3'
PTK2B	forward 5' AGTATCCATCTCCCGTTAACTCA 3'<br>reverse 5' CATCAGTCCGGTCCAGGTTAG 3'
EFNA1	forward 5' CATCTGTCCGCACTATGAAGATCAC 3'<br>reverse 5' ATGCTATGTAGAACCCGCACCT 3'
MNT	forward 5' CTTTGAGACCCTGAAGCGGAAC 3'<br>reverse 5' CTCCATATCCTCGTCTATGTTGTCCT 3'

LACTB	forward 5' GCTTCGCCAGAGCCATCGAG 3'<br>reverse 5' CCCACAATTTGGCAAGAGCAACC 3'
IFI16	forward 5' TTTCGAAGATATACCAACGC 3'<br>reverse 5' TCCTCTTTCTTGATAGGGCTG 3'
FADD	forward 5' CTGACCGAGCTCAAGTTCCTATGCC 3'<br>reverse 5' GTCACGGGCCTGCTGAACCTC 3'
ZFP36	forward 5' CTCATGGCCAACCGTTACAC 3'<br>reverse 5' ACTCAGTCCCTCCATGGTCG 3'
EEF1A1	forward 5' TACAACCCCGACACAGTAGCA 3'<br>reverse 5' TTGAAGCCCACATTGTCCCC 3'
MXI1	forward 5' CTGCGCCTTTGTTTAGAACGCTT 3'<br>reverse 5' CTGGCACTGGAGTAACCCTCGTCA 3'
BAX	forward 5' GTCCACCAAGAAGCTGAGCGAGT 3'<br>reverse 5' CTCCCGCCACAAAGATGGTCAC 3'
CIC	forward 5' AGGAGAAGCAGAAGTACCACGAC 3'<br>reverse 5' ATGCAGGGCCAGACACCATC 3'
CBLC	forward 5' GCCCACACCTTCTGGAGGGAAAGTTGC 3'<br>reverse 5' CTGAAAGAGCCTGGTGAAGACGTCGAAC 3'
PML	forward 5' CCCGCCCTGGATAACGTCT 3'<br>reverse 5' CTCCTGTCGCTGCTGGATCTCT 3'

Table S1. Antibodies and PCR primers. The source and catalog number of each antibody and the forward and reverse primer sequences for each target gene are listed.
